# Bis(μ_2_-4,7-dimethyl-4,7-diazadecane-1,10-dithiolato)trinickel(II) bis(perchlorate)

**DOI:** 10.1107/S1600536812006034

**Published:** 2012-02-17

**Authors:** Masakazu Hirotsu, Naoto Kuwamura, Isamu Kinoshita, Masaaki Kojima, Yuzo Yoshikawa

**Affiliations:** a Graduate School of Science, Osaka City University, Sumiyoshi-ku, Osaka 558-8585, Japan; b Department of Chemistry, Faculty of Science, Okayama University, Tsushima, Okayama 700-8530, Japan

## Abstract

In the title compound, [Ni_3_(C_10_H_22_N_2_S_2_)_2_](ClO_4_)_2_, the complex cation consists of a nickel(II) ion and two [Ni(C_10_H_22_N_2_S_2_)] units with an N_2_S_2_ tetra­dentate ligand, 3,3′-[1,2-ethane­diylbis(methyl­imino)]bis­(1-propane­thiol­ate). The central Ni^II^ ion is located on a crystallographic inversion centre and is bound to the four S atoms of the two [Ni(C_10_H_22_N_2_S_2_)] units to form a linear sulfur-bridged trimetallic moiety. The dihedral angle between the central NiS_4_ plane and the terminal NiN_2_S_2_ plane is 145.71 (5)°. In the [Ni(C_10_H_22_N_2_S_2_)] unit, the two methyl groups on the chelating N atoms are *cis* to each other, and the two six-membered *N*,*S*-chelate rings adopt a chair conformation. The Ni—S bond lengths and the S—Ni—S bite angles in the central NiS_4_ group are similar to those in the [Ni(C_10_H_22_N_2_S_2_)] unit.

## Related literature

For general background, see: Konno *et al.* (2000 ▶); Konno (2004 ▶); Igashira-Kamiyama & Konno (2011 ▶). For related structures, see: Grapperhaus *et al.* (2007 ▶); Turner *et al.* (1990 ▶).
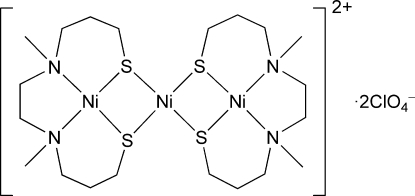


## Experimental

### 

#### Crystal data

[Ni_3_(C_10_H_22_N_2_S_2_)_2_](ClO_4_)_2_
*M* *_r_* = 843.83
Monoclinic, 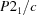

*a* = 8.0253 (19) Å
*b* = 16.208 (4) Å
*c* = 12.807 (3) Å
β = 105.033 (6)°
*V* = 1608.8 (7) Å^3^
*Z* = 2
Mo *K*α radiation
μ = 2.21 mm^−1^
*T* = 123 K
0.24 × 0.24 × 0.17 mm

#### Data collection

Rigaku AFC7 (Mercury CCD) diffractometer
Absorption correction: multi-scan (*REQAB*; Jacobson 1998 ▶) *T*_min_ = 0.619, *T*_max_ = 0.705
15030 measured reflections
3626 independent reflections
3391 reflections with *F* ^2^ > 2.0σ(*F* ^2^)
*R*_int_ = 0.027

#### Refinement

*R*[*F* ^2^ > 2σ(*F* ^2^)] = 0.035
*wR*(*F* ^2^) = 0.082
*S* = 1.04
3626 reflections
235 parameters
H atoms treated by a mixture of independent and constrained refinement
Δρ_max_ = 1.71 e Å^−3^
Δρ_min_ = −0.70 e Å^−3^

### 

Data collection: *CrystalClear* (Rigaku, 2007 ▶); cell refinement: *CrystalClear*; data reduction: *CrystalClear*; program(s) used to solve structure: *SHELXS97* (Sheldrick, 2008 ▶); program(s) used to refine structure: *SHELXL97* (Sheldrick, 2008 ▶); molecular graphics: *ORTEP-3 for Windows* (Farrugia, 1997 ▶); software used to prepare material for publication: *WinGX* (Farrugia, 1999 ▶) and *publCIF* (Westrip, 2010 ▶).

## Supplementary Material

Crystal structure: contains datablock(s) global, I. DOI: 10.1107/S1600536812006034/fj2508sup1.cif

Structure factors: contains datablock(s) I. DOI: 10.1107/S1600536812006034/fj2508Isup2.hkl

Additional supplementary materials:  crystallographic information; 3D view; checkCIF report

## Figures and Tables

**Fig. 1.**
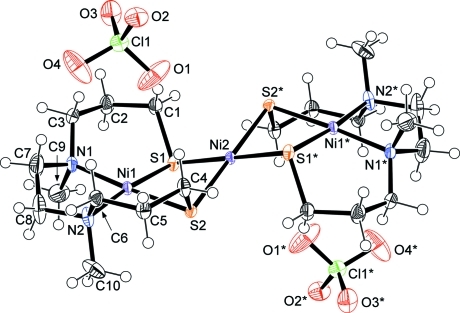
The molecular structure of (I) with numbering scheme. Displacement ellipsoids are drawn at the 50% probability level.

## supplementary crystallographic information

### Comment

Thiolate ligands have a high propensity to bridge transition metal ions and form sulfur-bridged polynuclear metal complexes. Metal complexes of polydentate ligands with thiolato-S atoms were used to construct supramolecular compounds (Konno *et al.*, 2000; Konno, 2004; Igashira-Kamiyama & Konno, 2011). Nickel(II) complexes containing diamine dithiolate ligands act as a bidentate-S,S metalloligand. The title compound [Ni{Ni(C_10_H_22_N_2_S_2_)}_2_](ClO_4_)_2_, (I), was synthesized by the reaction of 3,3'-[1,2-ethanediylbis(methylimino)]bis(1-propanethiol) with nickel(II) acetate tetrahydrate. The corresponding mononuclear nickel(II) complex [Ni(C_10_H_22_N_2_S_2_)], (II), which has a five-membered *N*,*N*- and two six-membered *N*,*S*-chelate rings, was synthesized and structurally characterized (Grapperhaus *et al.*, 2007). On the other hand, a mononuclear nickel(II) complex of 2,2'-[1,2-ethanediylbis(methylimino)]bis(ethanethiolate) (L), [Ni(L)], reacts with nickel(II) chloride to afford a sulfur-bridged trinuclear complex, [Ni{Ni(L)}_2_]Cl_2_, (III), in which the *S*,*N*,*N*,*S*-tetradentate ligand L forms five-membered *N*,*N*- and *N*,*S*-chelate rings (Turner *et al.*, 1990). The trinuclear complex (III) has a chair structure consisting of a central NiS_4_ and two terminal NiN_2_S_2_ planes, where the dihedral angle between the NiS_4_ and NiN_2_S_2_ planes is 107.84 (7)°. In this report, we discuss the structure of the new S-bridged Ni^II^_3_ complex (I), in which the size of the N,S-chelate rings is larger than that of (III).

The title compound (I) is composed of a complex cation, [Ni{Ni(C_10_H_22_N_2_S_2_)}_2_]^2+^, containing two N_2_S_2_ tetradentate ligands, 3,3'-[1,2-ethanediylbis(methylimino)]bis(1-propanethiolate), and two perchlorate anions (Fig. 1). The complex cation consists of a nickel(II) ion and two mononuclear [Ni(C_10_H_22_N_2_S_2_)] complex units, and the overall structure is similar to that of (III). The central Ni atom is located on a crystallographic inversion center and is surrounded by four S atoms of the two planar [Ni(C_10_H_22_N_2_S_2_)] units. The NiS_4_ structure is also planar. The structural parameters of the [Ni(C_10_H_22_N_2_S_2_)] unit in (I) are quite similar to those of the mononuclear complex (II). However, two methyl groups on the chelating N atoms of (I) are in a *cis* position to each other, while those of (II) are in a *trans* position. The dihedral angle between the NiS_4_ and NiN_2_S_2_ planes is 145.71 (5)°, which is significantly larger than that of (III) with five-membered N,S-chelate rings. Furthermore, the Ni—S—Ni angles (92.46 (3)°, 92.32 (2)°) and the Ni···Ni distance (3.1518 (6) Å) in (I) are larger than those in (III) (77.71 (4)°, 78.10 (4)°, 2.748 (1) Å). These results suggest that the chelate ring size of polydentate thiolate ligands largely affects the structure of S-bridged polynuclear metal complexes.

### Experimental

For the preparation of 3,3'-[1,2-ethanediylbis(methylimino)]bis(1-propanol), a solution of 3-bromo-1-propanol (11.66 g, 84 mmol) in CH_2_Cl_2_ (30 ml) was added dropwise to a solution of *N*,*N*'-dimethylethylenediamine (3.53 g, 40 mmol) and *N*,*N*-diisopropylethylamine (10.83 g, 84 mmol) in CH_2_Cl_2_ (20 ml). The solution was stirred for 31 h at room temperature. An aqueous solution of NaOH (4 mol dm^-3^, 50 ml) was added. The product was extracted with CH_2_Cl_2_ (300 ml). After removing the solvent, distillation under reduced pressure gave a colorless oil of 3,3'-[1,2-ethanediylbis(methylimino)]bis(1-propanol) (2.85 g, 35%). ^1^H NMR (270 MHz, CDCl_3_) δ 1.58–1.68 (m, 4H), 2.18 (s, 6H, C*H*_3_), 2.43 (s, 4H, NC*H*_2_C*H*_2_N), 2.49 (t, *J* = 6.3 Hz, 4H), 3.67 (t, *J* = 5.4 Hz, 4H), 5.20 (s, br, 2H).

For the preparation of 3,3'-[1,2-ethanediylbis(methylimino)]bis(1-propanethiol), a mixture of 3,3'-[1,2-ethanediylbis(methylimino)]bis(1-propanol) (0.82 g, 4.0 mmol), 47% HBr aq. (11 ml, 103 mmol), and thiourea (0.76 g, 10 mmol) was refluxed for 24 h. An aqueous solution of NaOH (2.5 mol dm^-3^, 52 ml) was added under N_2_, and the suspension was refluxed for 5 h under N_2_. The produced oil was extracted with diethyl ether (100 ml). The solution was adjusted to pH 8–9 with an aqueous HCl solution (2 mol dm^-3^), and the product was extracted with diethyl ether (200 ml). The combined extracts were dried over Na_2_SO_4_, and the solvent was removed by evaporation to afford a pale yellow oil (0.77 g, 81%). ^1^H NMR (270 MHz, CDCl_3_) δ 1.70–1.82 (m, 4H), 2.21 (s, 6H, C*H*_3_), 2.45 (s, 4H, NC*H*_2_C*H*_2_N), 2.39–2.58 (m, 8H).

For the synthesis of the title compound (I), a solution of 3,3'-[1,2-ethanediylbis(methylimino)]bis(1-propanethiol) (0.71 g, 3.0 mmol) in methanol (10 ml) was added to a suspension of nickel(II) acetate tetrahydrate (1.50 g, 6.0 mmol) in methanol (20 ml). The resulting dark brown suspension was stirred for 5 min, and then sodium perchlorate monohydrate (0.85 g, 6.0 mmol) was added. After stirring for 30 min, brown precipitate was filtered and washed with MeOH, H_2_O, and then MeOH. The brown solid of (I) was dried under reduced pressure over P_4_O_10_ (0.44 g, 35%). Red crystals suitable for X-ray analysis were obtained by heating a solution of (I) in *N*,*N*-dimethylformamide (DMF). Anal. Calcd for C_20_H_44_Cl_2_N_4_Ni_3_O_8_S_4_: C, 28.47; H, 5.26; N, 6.64%. Found: C, 28.63; H, 5.03; N, 6.68%. ^1^H NMR (300 MHz, dimethylsulfoxide-*d*_6_): δ 1.41–1.70 (m, br, 8H), 1.87–2.03 (m, br, 4H), 2.07–2.29 (m, br, 4H), 2.36–2.48 (m, br, 4H), 2.46 (s, 12H, C*H*_3_), 2.59–2.86 (m, br, 8H), 3.47–3.66 (m, br, 4H). ^13^C NMR (75.5 MHz, dimethylsulfoxide-*d*_6_): δ 24.0, 26.0, 45.3, 58.7, 60.1. UV-Vis (DMF): λ/nm (ε/dm^3^ mol^-1^ cm^-1^), 334 (14800), 397 (8730), 480 (1900, sh). Cyclic voltammogram (solvent, DMF; concentration, 5.0 × 10 ^-4^ mol dm^-3^; supporting electrolyte, 0.1 mol dm^-3^ Bu_4_NPF_6_; working electrode, 0.02 cm^2^ Pt disk electrode; scan rate, 100 mV/s): *E*/V (*versus* ferrocenium/ferrocene); *E*_pc_, -1.95 (*i*_pc_ = 1.7 µA); *E*_1/2_, -1.59 (Δ*E*_p_ = 69 mV, *i*_pa_/*i*_pc_ = 0.59, *i*_pc_ = 1.6 µA).

### Refinement

All non-H atoms were refined anisotropically. H atoms on the N,S-chelate rings were located in a difference Fourier map and were refined isotropically. All other H atoms were located on calculated positions with C—H(methylene) = 0.99 Å and C—H(methyl) = 0.98 Å, and were refined using a riding model with *U*_iso_(H) = 1.2*U*_eq_(C).

### Crystal data

**Table:**

[Ni_3_(C_10_H_22_N_2_S_2_)_2_](ClO_4_)_2_	*F*(000) = 876.00
*M**_r_* = 843.83	*D*_x_ = 1.742 Mg m^−^^3^
Monoclinic, *P*2_1_/*c*	Mo *K*α radiation, λ = 0.71075 Å
Hall symbol: -P 2ybc	Cell parameters from 7222 reflections
*a* = 8.0253 (19) Å	θ = 4.1–27.5°
*b* = 16.208 (4) Å	µ = 2.21 mm^−^^1^
*c* = 12.807 (3) Å	*T* = 123 K
β = 105.033 (6)°	Prism, red
*V* = 1608.8 (7) Å^3^	0.24 × 0.24 × 0.17 mm
*Z* = 2	

### Data collection

**Table:**

Rigaku AFC7 (Mercury CCD) diffractometer	3626 independent reflections
Radiation source: rotating anode X-ray tube	3391 reflections with *F*^2^ > 2.0σ(*F*^2^)
Graphite monochromator	*R*_int_ = 0.027
Detector resolution: 7.31 pixels mm^-1^	θ_max_ = 27.5°, θ_min_ = 4.1°
ω scans	*h* = −10→10
Absorption correction: multi-scan (REQAB; Jacobson, 1998)	*k* = −21→20
*T*_min_ = 0.619, *T*_max_ = 0.705	*l* = −13→16
15030 measured reflections	

### Refinement

**Table:**

Refinement on *F*^2^	0 restraints
Least-squares matrix: full	H atoms treated by a mixture of independent and constrained refinement
*R*[*F*^2^ > 2σ(*F*^2^)] = 0.035	*w* = 1/[σ^2^(*F*_o_^2^) + (0.0319*P*)^2^ + 3.9221*P*] where *P* = (*F*_o_^2^ + 2*F*_c_^2^)/3
*wR*(*F*^2^) = 0.082	(Δ/σ)_max_ = 0.002
*S* = 1.04	Δρ_max_ = 1.71 e Å^−^^3^
3626 reflections	Δρ_min_ = −0.70 e Å^−^^3^
235 parameters	

### Special details

**Table:**

Refinement. Refinement was performed using all reflections. The weighted *R*-factor (*wR*) and goodness of fit (*S*) are based on *F*^2^. *R*-factor (gt) are based on *F*. The threshold expression of *F*^2^ > 2.0 σ(*F*^2^) is used only for calculating *R*-factor (gt).

### Fractional atomic coordinates and isotropic or equivalent isotropic displacement parameters (Å^2^)

**Table:**

	*x*	*y*	*z*	*U*_iso_*/*U*_eq_	
Ni1	0.04531 (4)	0.626726 (19)	0.82272 (2)	0.01439 (10)	
Ni2	0.0000	0.5000	1.0000	0.01306 (11)	
Cl1	0.28814 (8)	0.39140 (4)	0.71998 (5)	0.02463 (15)	
S1	−0.16594 (8)	0.55517 (4)	0.85310 (5)	0.01655 (13)	
S2	0.14612 (8)	0.61385 (4)	0.99699 (5)	0.01539 (13)	
O1	0.2236 (4)	0.4191 (2)	0.8082 (2)	0.0720 (11)	
O2	0.4299 (3)	0.33683 (14)	0.76150 (19)	0.0357 (5)	
O3	0.1505 (3)	0.35038 (17)	0.64509 (18)	0.0431 (6)	
O4	0.3425 (4)	0.46058 (19)	0.6684 (3)	0.0680 (10)	
N1	−0.0478 (3)	0.63136 (14)	0.66462 (17)	0.0224 (5)	
N2	0.2398 (3)	0.69848 (15)	0.80846 (18)	0.0228 (5)	
C1	−0.2110 (4)	0.47418 (18)	0.7507 (2)	0.0241 (6)	
C2	−0.2552 (4)	0.5093 (2)	0.6370 (2)	0.0286 (6)	
C3	−0.1047 (4)	0.55199 (17)	0.6077 (2)	0.0222 (5)	
C4	0.3641 (3)	0.57575 (18)	1.0035 (2)	0.0212 (5)	
C5	0.4731 (4)	0.63872 (18)	0.9633 (2)	0.0225 (5)	
C6	0.4124 (3)	0.65719 (17)	0.8436 (2)	0.0202 (5)	
C7	0.1093 (5)	0.6568 (3)	0.6256 (3)	0.0433 (9)	
C8	0.2043 (5)	0.7223 (3)	0.6932 (3)	0.0433 (9)	
C9	−0.1854 (6)	0.6932 (2)	0.6355 (3)	0.0478 (10)	
C10	0.2471 (4)	0.77787 (18)	0.8711 (3)	0.0356 (7)	
H1A	−0.308 (5)	0.443 (2)	0.762 (3)	0.030 (9)*	
H1B	−0.117 (5)	0.443 (2)	0.759 (3)	0.028 (9)*	
H2A	−0.353 (4)	0.549 (2)	0.629 (3)	0.023 (8)*	
H2B	−0.284 (5)	0.464 (2)	0.587 (3)	0.038 (10)*	
H3A	−0.127 (5)	0.562 (2)	0.530 (3)	0.033 (9)*	
H3B	−0.005 (4)	0.514 (2)	0.624 (3)	0.024 (8)*	
H4A	0.413 (4)	0.562 (2)	1.076 (3)	0.027 (8)*	
H4B	0.353 (4)	0.526 (2)	0.960 (3)	0.022 (8)*	
H5A	0.477 (4)	0.688 (2)	1.004 (2)	0.017 (7)*	
H5B	0.589 (5)	0.617 (2)	0.974 (3)	0.027 (9)*	
H6A	0.498 (4)	0.6915 (19)	0.822 (2)	0.020 (7)*	
H6B	0.403 (4)	0.6070 (19)	0.800 (2)	0.017 (7)*	
H7A	0.1858	0.6086	0.6278	0.052*	
H7B	0.0711	0.6762	0.5497	0.052*	
H8A	0.1359	0.7739	0.6806	0.052*	
H8B	0.3143	0.7325	0.6740	0.052*	
H9A	−0.1460	0.7449	0.6735	0.072*	
H9B	−0.2876	0.6732	0.6562	0.072*	
H9C	−0.2146	0.7028	0.5573	0.072*	
H10A	0.2703	0.7655	0.9485	0.053*	
H10B	0.1364	0.8067	0.8472	0.053*	
H10C	0.3393	0.8130	0.8584	0.053*	

### Atomic displacement parameters (Å^2^)

**Table:**

	*U*^11^	*U*^22^	*U*^33^	*U*^12^	*U*^13^	*U*^23^
Ni1	0.01653 (17)	0.01475 (17)	0.01300 (16)	0.00004 (11)	0.00584 (12)	0.00248 (11)
Ni2	0.0134 (2)	0.0146 (2)	0.0115 (2)	−0.00204 (16)	0.00396 (15)	0.00100 (15)
Cl1	0.0182 (3)	0.0289 (3)	0.0237 (3)	0.0002 (2)	−0.0003 (2)	−0.0014 (3)
S1	0.0151 (3)	0.0209 (3)	0.0145 (3)	0.0007 (2)	0.0053 (2)	0.0034 (2)
S2	0.0182 (3)	0.0161 (3)	0.0133 (3)	−0.0033 (2)	0.0064 (2)	−0.0009 (2)
O1	0.0498 (17)	0.120 (3)	0.0414 (15)	0.0461 (19)	0.0037 (13)	−0.0256 (17)
O2	0.0267 (11)	0.0350 (12)	0.0436 (13)	0.0125 (9)	0.0061 (9)	−0.0016 (10)
O3	0.0362 (13)	0.0549 (15)	0.0302 (12)	−0.0205 (12)	−0.0056 (10)	0.0011 (11)
O4	0.0394 (15)	0.0534 (17)	0.098 (2)	−0.0159 (13)	−0.0057 (15)	0.0363 (17)
N1	0.0326 (13)	0.0209 (11)	0.0140 (10)	−0.0057 (9)	0.0066 (9)	0.0031 (8)
N2	0.0195 (11)	0.0279 (12)	0.0220 (11)	0.0008 (9)	0.0071 (9)	0.0125 (9)
C1	0.0269 (15)	0.0270 (14)	0.0168 (12)	−0.0116 (12)	0.0028 (10)	0.0000 (11)
C2	0.0315 (16)	0.0348 (16)	0.0161 (12)	−0.0104 (13)	0.0001 (11)	0.0015 (12)
C3	0.0282 (14)	0.0242 (14)	0.0146 (11)	0.0002 (11)	0.0065 (10)	0.0005 (10)
C4	0.0160 (12)	0.0272 (14)	0.0194 (12)	−0.0016 (10)	0.0028 (10)	0.0074 (11)
C5	0.0178 (13)	0.0273 (14)	0.0221 (13)	−0.0043 (11)	0.0045 (10)	0.0030 (11)
C6	0.0194 (13)	0.0225 (13)	0.0210 (12)	−0.0005 (10)	0.0095 (10)	0.0016 (10)
C7	0.052 (2)	0.055 (2)	0.0265 (15)	−0.0176 (18)	0.0173 (15)	0.0042 (15)
C8	0.0329 (17)	0.068 (2)	0.0264 (15)	−0.0106 (17)	0.0029 (13)	0.0233 (16)
C9	0.076 (3)	0.0329 (18)	0.0271 (16)	0.0233 (18)	−0.0007 (16)	0.0034 (14)
C10	0.0289 (16)	0.0184 (14)	0.064 (2)	−0.0007 (12)	0.0207 (15)	0.0058 (14)

### Geometric parameters (Å, º)

**Table:**

Ni1—N1	1.969 (2)	C4—H4B	0.98 (3)
Ni1—N2	1.993 (2)	C5—C6	1.513 (4)
Ni1—S1	2.1709 (8)	C5—H5A	0.95 (3)
Ni1—S2	2.1777 (8)	C5—H5B	0.97 (4)
Ni2—S1	2.1938 (7)	C6—N2	1.498 (3)
Ni2—S2	2.1921 (7)	C6—H6A	0.98 (3)
Cl1—O1	1.432 (3)	C6—H6B	0.98 (3)
Cl1—O2	1.430 (2)	C7—C8	1.454 (5)
Cl1—O3	1.425 (2)	C7—N1	1.529 (4)
Cl1—O4	1.426 (3)	C7—H7A	0.9900
C1—C2	1.518 (4)	C7—H7B	0.9900
C1—S1	1.824 (3)	C8—N2	1.481 (4)
C1—H1A	0.97 (4)	C8—H8A	0.9900
C1—H1B	0.89 (4)	C8—H8B	0.9900
C2—C3	1.520 (4)	C9—N1	1.466 (4)
C2—H2A	1.00 (3)	C9—H9A	0.9800
C2—H2B	0.97 (4)	C9—H9B	0.9800
C3—N1	1.491 (3)	C9—H9C	0.9800
C3—H3A	0.98 (4)	C10—N2	1.510 (4)
C3—H3B	0.99 (3)	C10—H10A	0.9800
C4—C5	1.519 (4)	C10—H10B	0.9800
C4—S2	1.836 (3)	C10—H10C	0.9800
C4—H4A	0.94 (3)	Ni1—Ni2	3.1518 (6)
			
N1—Ni1—N2	88.90 (9)	C3—C2—H2A	109.8 (19)
N1—Ni1—S1	95.68 (7)	C1—C2—H2B	108 (2)
N2—Ni1—S1	174.27 (7)	C3—C2—H2B	105 (2)
N1—Ni1—S2	176.66 (7)	H2A—C2—H2B	112 (3)
N2—Ni1—S2	93.34 (7)	N1—C3—H3A	108 (2)
S1—Ni1—S2	82.24 (3)	C2—C3—H3A	112 (2)
S1—Ni2—S2	81.39 (3)	N1—C3—H3B	107.9 (19)
S1—Ni2—S2^i^	98.61 (3)	C2—C3—H3B	108.7 (19)
Ni1—S1—C1	105.91 (10)	H3A—C3—H3B	104 (3)
Ni2—S1—C1	106.63 (10)	C5—C4—H4A	112 (2)
Ni1—S2—C4	100.09 (9)	S2—C4—H4A	106 (2)
Ni2—S2—C4	102.93 (9)	C5—C4—H4B	110.2 (19)
Ni1—S1—Ni2	92.46 (3)	S2—C4—H4B	107.8 (19)
Ni1—S2—Ni2	92.32 (2)	H4A—C4—H4B	109 (3)
O3—Cl1—O4	109.37 (17)	C6—C5—H5A	110.1 (18)
O3—Cl1—O2	111.38 (16)	C4—C5—H5A	108.8 (18)
O4—Cl1—O2	110.42 (16)	C6—C5—H5B	105 (2)
O3—Cl1—O1	107.56 (19)	C4—C5—H5B	109 (2)
O4—Cl1—O1	109.6 (2)	H5A—C5—H5B	110 (3)
O2—Cl1—O1	108.46 (16)	N2—C6—H6A	108.8 (18)
C2—C1—S1	111.9 (2)	C5—C6—H6A	109.7 (18)
C1—C2—C3	113.9 (2)	N2—C6—H6B	106.0 (18)
N1—C3—C2	115.7 (2)	C5—C6—H6B	111.5 (18)
C5—C4—S2	112.6 (2)	H6A—C6—H6B	105 (2)
C6—C5—C4	114.5 (2)	C8—C7—H7A	109.5
N2—C6—C5	115.0 (2)	N1—C7—H7A	109.5
C8—C7—N1	110.6 (3)	C8—C7—H7B	109.5
C7—C8—N2	109.8 (3)	N1—C7—H7B	109.5
C9—N1—C3	110.5 (2)	H7A—C7—H7B	108.1
C9—N1—C7	111.3 (3)	C7—C8—H8A	109.7
C3—N1—C7	104.3 (2)	N2—C8—H8A	109.7
C9—N1—Ni1	110.34 (19)	C7—C8—H8B	109.7
C3—N1—Ni1	117.14 (16)	N2—C8—H8B	109.7
C7—N1—Ni1	102.81 (18)	H8A—C8—H8B	108.2
C8—N2—C6	109.9 (2)	N1—C9—H9A	109.5
C8—N2—C10	106.2 (3)	N1—C9—H9B	109.5
C6—N2—C10	108.4 (2)	H9A—C9—H9B	109.5
C8—N2—Ni1	106.90 (19)	N1—C9—H9C	109.5
C6—N2—Ni1	113.38 (16)	H9A—C9—H9C	109.5
C10—N2—Ni1	111.83 (17)	H9B—C9—H9C	109.5
C2—C1—H1A	110 (2)	N2—C10—H10A	109.5
S1—C1—H1A	106 (2)	N2—C10—H10B	109.5
C2—C1—H1B	108 (2)	H10A—C10—H10B	109.5
S1—C1—H1B	109 (2)	N2—C10—H10C	109.5
H1A—C1—H1B	112 (3)	H10A—C10—H10C	109.5
C1—C2—H2A	108.7 (18)	H10B—C10—H10C	109.5
			
S1—C1—C2—C3	67.6 (3)	S1—Ni1—Ni2—S2^i^	37.83 (4)
C1—C2—C3—N1	−69.6 (3)	S2—Ni1—Ni2—S2^i^	180.0
S2—C4—C5—C6	−67.3 (3)	N1—Ni1—Ni2—S2	177.99 (10)
C4—C5—C6—N2	65.5 (3)	N2—Ni1—Ni2—S2	30.68 (10)
N1—C7—C8—N2	51.0 (4)	S1—Ni1—Ni2—S2	−142.17 (4)
C2—C3—N1—C9	−63.8 (3)	N1—Ni1—Ni2—S1^i^	140.16 (10)
C2—C3—N1—C7	176.5 (3)	N2—Ni1—Ni2—S1^i^	−7.16 (10)
C2—C3—N1—Ni1	63.7 (3)	S1—Ni1—Ni2—S1^i^	180.0
C8—C7—N1—C9	74.6 (4)	S2—Ni1—Ni2—S1^i^	−37.83 (4)
C8—C7—N1—C3	−166.2 (3)	N1—Ni1—Ni2—S1	−39.84 (10)
C8—C7—N1—Ni1	−43.5 (3)	N2—Ni1—Ni2—S1	172.84 (10)
C7—C8—N2—C6	92.5 (3)	S2—Ni1—Ni2—S1	142.17 (4)
C7—C8—N2—C10	−150.5 (3)	C2—C1—S1—Ni1	−57.8 (2)
C7—C8—N2—Ni1	−31.0 (4)	C2—C1—S1—Ni2	−155.3 (2)
C5—C6—N2—C8	173.2 (3)	N1—Ni1—S1—C1	43.83 (13)
C5—C6—N2—C10	57.6 (3)	S2—Ni1—S1—C1	−133.54 (11)
C5—C6—N2—Ni1	−67.2 (3)	Ni2—Ni1—S1—C1	−108.06 (11)
C9—N1—Ni1—N2	−98.6 (2)	N1—Ni1—S1—Ni2	151.89 (7)
C3—N1—Ni1—N2	133.8 (2)	S2—Ni1—S1—Ni2	−25.48 (2)
C7—N1—Ni1—N2	20.1 (2)	S2^i^—Ni2—S1—C1	−47.23 (10)
C9—N1—Ni1—S1	77.9 (2)	S2—Ni2—S1—C1	132.77 (10)
C3—N1—Ni1—S1	−49.7 (2)	Ni1—Ni2—S1—C1	107.41 (10)
C7—N1—Ni1—S1	−163.34 (19)	S2^i^—Ni2—S1—Ni1	−154.64 (2)
C9—N1—Ni1—Ni2	104.5 (2)	S2—Ni2—S1—Ni1	25.36 (2)
C3—N1—Ni1—Ni2	−23.1 (2)	C5—C4—S2—Ni1	64.9 (2)
C7—N1—Ni1—Ni2	−136.74 (18)	C5—C4—S2—Ni2	159.63 (18)
C8—N2—Ni1—N1	4.8 (2)	N2—Ni1—S2—C4	−54.60 (12)
C6—N2—Ni1—N1	−116.41 (18)	S1—Ni1—S2—C4	129.07 (10)
C10—N2—Ni1—N1	120.7 (2)	Ni2—Ni1—S2—C4	103.57 (10)
C8—N2—Ni1—S2	−177.6 (2)	N2—Ni1—S2—Ni2	−158.17 (7)
C6—N2—Ni1—S2	61.11 (17)	S1—Ni1—S2—Ni2	25.50 (2)
C10—N2—Ni1—S2	−61.82 (19)	S1^i^—Ni2—S2—C4	53.82 (9)
C8—N2—Ni1—Ni2	161.56 (19)	S1—Ni2—S2—C4	−126.18 (9)
C6—N2—Ni1—Ni2	40.3 (2)	Ni1—Ni2—S2—C4	−100.91 (9)
C10—N2—Ni1—Ni2	−82.6 (2)	S1^i^—Ni2—S2—Ni1	154.73 (2)
N1—Ni1—Ni2—S2^i^	−2.01 (10)	S1—Ni2—S2—Ni1	−25.27 (2)
N2—Ni1—Ni2—S2^i^	−149.32 (10)		

Symmetry code: (i) −*x*, −*y*+1, −*z*+2.
